# First Glimpse into the Genomic Characterization of People from the Imperial Roman Community of Casal Bertone (Rome, First–Third Centuries AD)

**DOI:** 10.3390/genes13010136

**Published:** 2022-01-13

**Authors:** Flavio De Angelis, Marco Romboni, Virginia Veltre, Paola Catalano, Cristina Martínez-Labarga, Valentina Gazzaniga, Olga Rickards

**Affiliations:** 1 Centre of Molecular Anthropology for Ancient DNA Studies, Department of Biology, University of Rome Tor Vergata, 00133 Rome, Italy; virginia.veltre@uniroma2.it (V.V.); martine@uniroma2.it (C.M.-L.); rickards@uniroma2.it (O.R.); 2 Department of Biology, University of Pisa, 56121 Pisa, Italy; marco.romboni@phd.unipi.it; 3 PhD Program in Evolutionary Biology and Ecology, Department of Biology, University of Rome Tor Vergata, 00133 Roma, Italy; 4 Former Servizio di Antropologia, Soprintendenza Speciale Archeologia, Belle Arti e Paesaggio di Roma, 00185 Roma, Italy; paola.catalano129@gmail.com; 5 Unità di Storia della Medicina e Bioetica, Sapienza University of Rome, 00185 Roma, Italy; valentina.gazzaniga@uniroma1.it

**Keywords:** Roman fullers, ancient DNA, children, metagenomes, microbes

## Abstract

This paper aims to provide a first glimpse into the genomic characterization of individuals buried in Casal Bertone (Rome, first–third centuries AD) to gain preliminary insight into the genetic makeup of people who lived near a tannery workshop, *fullonica.* Therefore, we explored the genetic characteristics of individuals who were putatively recruited as fuller workers outside the Roman population. Moreover, we identified the microbial communities associated with humans to detect microbes associated with the unhealthy environment supposed for such a workshop. We examined five individuals from Casal Bertone for ancient DNA analysis through whole-genome sequencing via a shotgun approach. We conducted multiple investigations to unveil the genetic components featured in the samples studied and their associated microbial communities. We generated reliable whole-genome data for three samples surviving the quality controls. The individuals were descendants of people from North African and the Near East, two of the main foci for tannery and dyeing activity in the past. Our evaluation of the microbes associated with the skeletal samples showed microbes growing in soils with waste products used in the tannery process, indicating that people lived, died, and were buried around places where they worked. In that perspective, the results represent the first genomic characterization of fullers from the past. This analysis broadens our knowledge about the presence of multiple ancestries in Imperial Rome, marking a starting point for future data integration as part of interdisciplinary research on human mobility and the bio-cultural characteristics of people employed in dedicated workshops.

## 1. Introduction

Virgil described the Romans as *gens togata*—people that wear the toga—in the Aeneid when referring to the Roman people but not to people who were not Roman citizens; thus, dressing attire was meaningful in ancient Rome [1,2,3].

The history of Roman fashion ran parallel with that of the arts and architecture: indeed, they inherited ideas from the Greeks, but the assimilation of people of different customs led to a change in the original style, which became complex and richly colored.

Thus, various treatments were developed to create attire that represented the wearer’s social position in a society where self-presentation mattered [4]. These procedures were progressively tuned in dedicated workshops such as *fullonicae*—ancient tanneries. *Fullonicae* were multifunctional facilities for textile processing. They were populated by fullers, whose guilds were funded by *Numa Pompilio* in the seventh century BCE, as noted in multiple epigraphic records throughout the Roman Empire.

Fulling is frequently mentioned in classical references, and this activity was considerably profitable, at least for the owners or supervisors [5].

People employed in a *fullonica* could lean on the walls between the tubs when treading the textiles stomped underfoot with alkaline substances such as human or animal urine in water (*Saltus fullonicus*) [6]. They were often slaves, though the social basis of the staff has been deeply questioned [7].

Only minor information about fullers’ origins and their social organization exists. Much evidence suggested that the ancient tanneries were widespread in the Roman Empire, with several foci located in the southern and eastern shores of the Mediterranean Sea [4,7,8]. Therefore, we aim to broaden our knowledge about the spread of these workshops, which could be related to the massive Rome-ward migration during the Imperial Age.

We hypothesize that people employed in a *fullonica* were related to immigrants to Rome, whose genetic legacy could be traced back to their former populations. Indeed, the genomic characterization of ancient Romans from Rome is increasingly showing a complex scenario [9,10], even though the data are still limited and rarely correlated with specific archeological interpretations for people to be considered allochthonous. Comparisons with available data for the inhabitants of Imperial Rome are meaningful in elucidating the genetic relationships across multiple human groups living in the capital of the Roman Empire. Thus far, an understanding of the demographics of different specialized workers during the Roman Empire could be pivotal in a proper reconstruction of the highly multi-ethnic urban landscape of Rome during the Imperial Age, paving the way to the effective identification of migration waves from all around the Roman Empire.

Furthermore, the classical sources gave clues about the fullers’ daily lives and social organization. The *fullo* of the Apuleius’ Metamorphoses (Met 9.25) lived and worked alongside their wives [7], suggesting that fullers had families in the immediate surroundings of their workshop, even accounting for the unhealthy environment characterizing *fullonicae*.

The harsh conditions of the workshop environment also likely affected the workers’ health. Indeed, classical sources stated that continuous immersion in the liquids used inevitably made fullers vulnerable to bacterial, viral, and fungal infections due to repeated exposure to urine and organic waste [5]. The remains of workshops dedicated to fulling are among the most significant material evidence for manufacturing activities in Roman towns [4]. The first Italian tannery discovered was excavated in Pompei in 1826 and was attributed to *Lucius Veranius Hypsaeus* [8]. However, notable findings were scattered throughout the Empire, and noteworthy were those discovered in Pompei, Ostia, Florence, Roman Asia Minor, and Roman Egypt [7,8].

**Figure 1:**
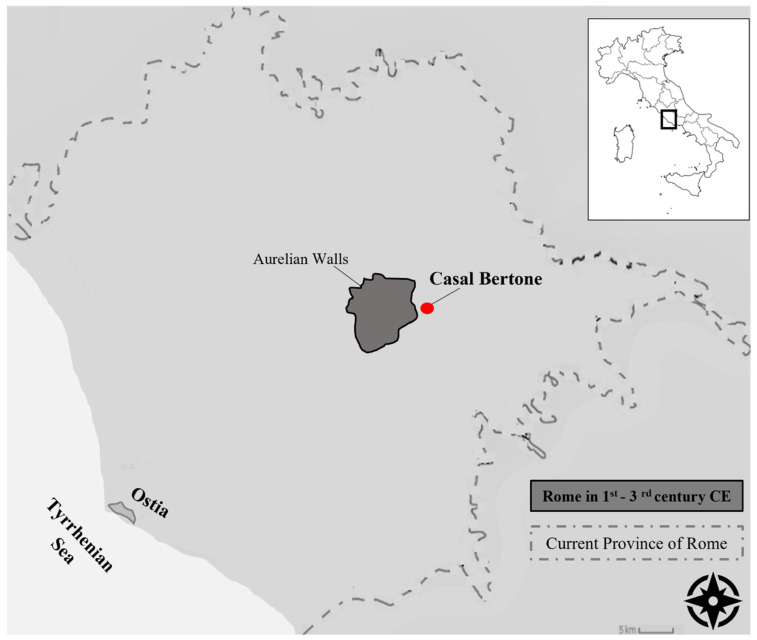
The red dot marks the geographic location of Casal Bertone. The dark area represents the Roman borders embodied by the Aurelian walls in the third century CE.

A 1255-square-yard tannery dated back to the first–third centuries was recently discovered in Casal Bertone, Rome (Figure 1), along with burial sites and a section of a Roman paved road. Notably, the highest number of *pilae fullonicae*—the individual workplace for a fuller—was herein discovered [8,11,12]. This workshop was located just outside the city—3.5 km away from the Aurelian walls—possibly to hide its unhealthy environment [7,13,14].

We were not able to identify any workers’ dwellings; however, a heterogeneous bi-ritual necropolis (with both inhumations and cremations) was discovered close to the factory, perhaps to accommodate burial requests by the people employed in the tannery and their families. Indeed, the skeletons presented biomechanical stress markers consistent with the multiple work stages described for such a workshop, even when considering the remains of children [15,16,17]. The funerary context was archaeologically subdivided into multiple sections: a mausoleum; a graveyard; and an area, named Area Q, contiguous to the production area. Even though each tomb had heterogeneous characteristics, they generally pertained to low social strata. Indeed, even though people were buried inside the mausoleum, often, they were in *formae*, where multiple layers of inhumations could be set [17]. The demographic profile of the areas led us to consider the mausoleum and necropolis communities as a single population [18]. Despite a remarkable difference in the distribution of multiple osteological stress markers, the social differentiation across the people buried in those different areas has not yet been verified. However, a tentative hypothesis suggests that the inhumations in the mausoleum are for extended familial clusters of people living in subtly better conditions [17]. Conversely, the demographic profile of Area Q is significantly dissimilar due to the substantial presence of child burials. This odd demographic profile was previously associated with the challenging lifestyles experienced by fuller heirs and the spread of infectious diseases [18].

People from all areas have been already characterized by osteological stress markers, demography, diet preferences, and mobility pattern reconstruction [11,12,13,14,15,16,17,18,19,20,21,22,23]; however, no genomic description has ever been attempted.

Moreover, as we generated genomic data using a shotgun sequencing approach, we tried to identify the microbial communities associated with humans to detect microbes related to the unhealthy environment supposed of a *fullonica*, which might be responsible for the massive death rate in children from the area.

Accordingly, this paper aims to take the first glimpse into the genomic characterization of individuals buried in Casal Bertone to gain preliminary insight into the genetic makeup of people who lived near these workshops. In that perspective, the results represent the first genomic characterization of fullers from the past, marking a starting point for ongoing data integration for interdisciplinary research on the bio-cultural characteristics of people living in ancient Rome.

## 2. Materials and Methods

### 2.1. Samples

We examined five Casal Bertone (CB) individuals for the genomic analysis (Table 1). They were all children, putatively extended families massively employed in the *fullonica* and living close to the workshop. F4_I and F9 were probably actively employed in the workshop as their bones were characterized by biomechanical stress markers [15]. Conversely, T9, T21, and T46 were vulnerable to the area’s environmental factors. The archaeological context and the typology of their funerary structures indicate that they could be dated back to the first–third centuries CE, even though no radiocarbon data are available. With the permissions obtained and due to the ongoing osteological evaluations, multiple bone districts were examined for genomic analysis. The primary inclusion criterion was macroscopic bone preservation. Each specimen was treated individually, and the sequences mapped to the human genome from a single individual were merged for further analysis to maximize the informativeness.

### 2.2. DNA Extraction and Library Preparation

The samples were processed in a clean laboratory of the Centre of Molecular Anthropology for Ancient DNA studies, University of Rome Tor Vergata (Italy). The facilities are exclusively set for ancient DNA recovery and processing. Entrance airlocks and a HEPA-filtered conditioning system grant a clean environment. All of the surfaces and equipment were cleaned with a sodium hypochlorite solution, rinsed with water, and dehydrated using ethanol. UV lighting ensures DNA decontamination of reagent solutions and surfaces, and all personnel wore protective equipment. Surgical blades were used to remove soil from the samples effectively, and the outer teeth surface was bleached with a clean wipe. The samples were UV-irradiated overnight at about 6 J/cm^2^ at 254 nm. The powder for DNA extraction was produced using a Dremel drill applying the lowest speed [24] and collected into 2 mL vials. The weight of the powder obtained varied between 0.08 and 0.1 g. The powder was incubated, rotating for 24 to 48 h at 37 °C in 1 mL of the extraction buffer—urea in EDTA (0.5 M) and 10 µL of proteinase K (20 mg/mL) [25]—and negative controls were set and maintained through the extraction protocol. The supernatant was collected and transferred to an Amicon Ultra-4 Centrifugal Filter Unit with Ultracel-30 from Millipore for spinning down to 100 mL. DNA was extracted and purified using MiniElute spin columns and Qiagen buffer and was stored at −20 °C. An Illumina double-stranded library was built from 20 mL of extracted DNA for each specimen [26]. We determined the number of indexing cycles using RT-PCR (ThermoFisher, Waltham, MA, USA). Indexing PCRs were set up in 25 mL with a final concentration of 1× Gold Buffer (ThermoFisher, Waltham, MA, USA), 2.5 mM Magnesium Chloride, 250 mM dNTP (each), 3 mL of DNA library, 0.2 mM for each indexing primer, and 0.1 U/mL of AmpliTaq Gold (ThermoFisher, Waltham, MA, USA). The cycling conditions for library amplification were 5 min at 95 °C; followed by cycles of 30 s at 95 °C, 30 s at 60 °C, and 45 s at 72 °C; and a final extension of 5 min at 72 °C. Each library was six-fold amplified along with PCR blanks. PCR products were pooled and purified with Agencourt AMPureXP beads (Beckman Coulter, Indianapolis, IN, USA). The concentration and size profiles of the purified library were detected using a Bioanalyzer 2100 through a High Sensitivity DNA chip (Agilent, Santa Clara, CA, USA). Equimolar concentration pools were submitted to whole-genome sequencing on the Illumina HiSeqX (2 × 150 bp) at Macrogene (Seoul, Korea).

### 2.3. Data Analysis

Paired-end reads were mapped to the human reference genome (GRCh37/hg19) as single-end reads using BWA mem algorithm v0.7.17 [27], previously trimming peculiar sequences as molecular barcodes and adapters using cutadapt v1.15 [28]. The trimming of 10 bases from each reduces the error rate due to deamination.

We merged the data obtained from multiple specimens of each sample with samtools merge [29] to increase the coverage and the robustness of the analysis. We removed duplicated sequences by identifying sets of reads with the same external coordinates after alignment to GRCh37/hg19, executing the rmdup function of samtools v1.7 [29]. PMDtools [30] was used to compute ancient DNA damage patterns and to identify degraded sequences that were unlikely to originate from modern contamination.

Each sequence was assigned a PMD score, for which positive values support the sequence being genuinely ancient. PMD scores > 2 allow for filtering ancient DNA molecules from the others. We assessed the authenticity and the quality of the suitable libraries using ANGSD software [31] to obtain a conservative estimate of contamination in the X-chromosome of males as they have only one X chromosome and are not expected to be polymorphic. The alignments were merged through the MergeSamFiles option, available in picardtools (https://broadinstitute.github.io/picard/, accessed on 5 August 2021). Qualimap [32] returned the basic descriptive statistics for each treated alignment. Samples’ variants were filtered to those present in the 1240K_Human Origins panel (release v42.4 597,573 SNPs, https://reich.hms.harvard.edu/datasets, accessed on 4 September 2021) to optimize the comparison between them, ancient, and modern published references. Furthermore, filters of a minimum base and mapping quality of 30 (–min-BQ and –min-MQ options) were used via samtools mpileup v1.7 [29]. Pseudo haploid genotypes were called by randomly choosing one allele from each site, using pileupCaller [33], to monitor the impact of reference bias on downstream analyses due to the well-known properties of ancient DNA (i.e., fragmented molecules and low-covered sequences) [34]. Principal component analysis (PCA) was performed using smartpca v0.13050 from EIGENSOFT package v1.6.4 [35]. The setting phase was planned by selecting the reference individuals—535 individuals from 29 modern populations—primarily related to three geographic macro-areas (southern Europe, the Middle East, and northern Africa) (Supplementary Table S1). Therefore, the samples studied were projected into the PC space through the lsqproject option, allowing for the handling of ancient samples with a considerable amount of missing data. Moreover, 148 individuals belonging to ancient populations covering almost the same regions (Supplementary Table S1) were also projected in the PCA, using the “shrinkmode: YES” option.

An ADMIXTURE analysis was carried out to reveal the genetic component of the studied samples. The variant panels were previously pruned of those in linkage disequilibrium through the PLINK 1.9 option –indep-pairwise 100 5 0.8, leading to 412,388 SNPs. We performed five replications with different random seeds for each K (from K2 to K6), using the –cv option with five-fold cross-validation and retaining the highest likelihood replication for each value of K [36]. We exploited pong v1.4.9, a freely available user-friendly graphical tool [37], to visualize the admixture proportion inferences. The “–s 0.95” parameter allowed us to collect the outputs into the same benchwork, showing them in a ranked order according to the number of runs supporting each estimation [38]. To further figure out the population relationships, we computed the outgroup f3 statistics. This parameter is a useful analytical tool for quantifying the shared drift between two groups (or individuals) by measuring the length of the shared branch between a pair of groups (i.e., shared genetic drift) from an outgroup on the phylogenetic tree. qp3Pop from ADMIXTOOLS v6.0 was used, and consequently, the analysis was computed considering the standard errors with a block jackknife [39]. The outgroup f3 statistic was solved in the form (Papuan; X, Y) on the customized 1240 K_HO panel restricted as described above. This strategy was pursued to account for the putative genetic similarities of people buried in CB with the African populations.

Similarly, to improve our analysis, we tested for differences among comparative populations through D-statistics in the form of D (Papuan, CB; Pop1, Pop2) [39] using an outgroup population (Papuan) that did not experience any post-divergence gene flow with the tested populations. The molecular sex of individuals was estimated using the Ry method [40]. The process divides the number of sequences mapped to the Y chromosome from those mapped to X and Y chromosomes. Raw data originating from the sequencing platform were also submitted to the PALEOMIX v1.2.14 bam pipeline [41]. This pipeline enabled us to set some customized options for mitogenome alignment against the revised Cambridge Reference Sequence (rCRS) (NC_012920.1). The resulting bam files were filtered, sorted, and indexed. Notably, contamination and postmortem damage estimations were performed by MapDamage 2.0 as part of this automated process. Varscan mpileup, Galaxy version 2.4.3.1 [42], with default parameters allowed us to obtain the consensus genotype using the pileup file as an input. The restored mitogenomes were submitted to the haplogroup inference analysis through the Haplogrep build on the Phylotree 17 classification algorithm [43]. The Y chromosome reads of each male individual were filtered out from the alignments using samtools v1.7. Leveraging the list of SNPs informative of the Y-Chr haplogroups, downloaded from the International Society of Genetic Genealogy website (https://isogg.org/tree/; Version 15.73, accessed on 11 July 2020), we called the genotype at these positions. Only reads showing minimum mapping and base quality scores of 30 were used. The Y chromosome haplogroups were assigned using yhaplo software v1.1.0 (https://github.com/23andMe/yhaplo, accessed on 16 September 2021) [44].

We used a pairwise mismatch on the pseudo haploid data (READ [45]) to identify putative genetic kinship relationships across individuals.

Furthermore, we ran HOME-BIO (sHOtgun MEtagenomic analysis of BIOlogical entities) to obtain an overview of the host-associated microbial communities for the analyzed samples. This all-in-one pipeline is a dockerized solution for analyzing shotgun datasets based on Kraken2 pre-built databases (virus, bacteria, and protozoa) and Kaiju for taxonomic classification [46]. We used only the metagenomic shotgun module of the two main modules (Metagenomic shotgun and Assembly de novo), preceded by the trimming step previously described for the human genomic analysis and a quality control step combining quality checks of the shotgun reads through FASTQC (https://www.bioinformatics.babraham.ac.uk/projects/fastqc/, accessed on 25 September 2021) and MultiQC [47]. We filtered out host reads that may interfere with the results after adapter trimming and removed low-quality reads [28] before aligning the references [48]. The selected module performs taxonomic profiling by classifying unmapped reads with Kraken2 [49] using the default confidence score threshold of 0.5 to define the quality of taxonomic classification. Bacterial, viral, and protozoal NCBI datasets built by Kraken2 and provided in the downloaded resource were used to maximize the information.

Even though HOME-BIO provided a Kaiju protein-level classification [50], we did not use this alignment. All of the classified entries were processed with Krona [51] for graphic visualization.

## 3. Results

### 3.1. Human Genetic Profiles

We generated whole-genome raw data for each specimen of the five individuals from Casal Bertone. Preliminary processes related to the data QC allowed us to collect alignment data for all of them, which returned at least 10,000 reads mapping to the human reference genome. Their mean coverages and descriptive statistics are presented in Table 2 and Supplementary Table S2.

**Figure 2:**
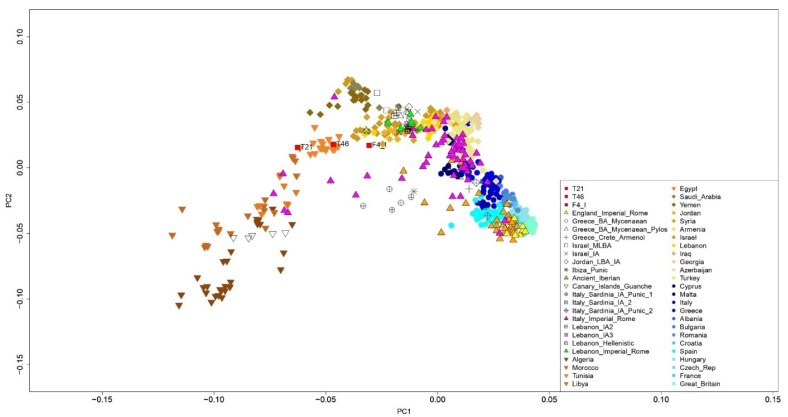
Principal component analysis (PCA) plot for the visualization of CB samples (labeled as red squares) and individuals from the Supplementary Table S1, using the “shrinkmode: YES” option.

PMDtools allows for the generation of a dataset of sequences that should be considered genuinely ancient. The endogenous DNA genomic coverage ranges between 0.0003 and 0.0562x, suggesting that the amount of DNA preservation was mined by taphonomy and the chemical-physical processes that occur in graves. Indeed, the humid environment characterizing the site was detrimental to the proper preservation of ancient DNA [52]. We performed principal component analysis (PCA) to compare the analyzed individuals with contemporary and modern populations. The samples from CB and 148 roughly coeval samples from topographically scattered areas were projected onto the genetic variation of 535 present-day individuals from the greater Mediterranean region (Figure 2).

Only three samples sharing more than 15,000 variants across the dataset were plotted (Table 2) and used for further analyses after checking the contamination rate (Supplementary Table S2). People from CB were genetically similar to North African and Middle Eastern individuals. Specifically, T21 and T46 were similar to present-day Egyptians, while F4_I was similar to Middle Eastern populations such as those found in Israel.

**Figure 3:**
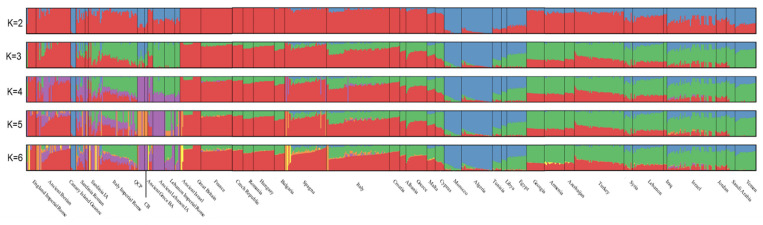
ADMIXTURE results from K = 2 to K = 6.

A model-based clustering (Figure 3) of selected present-day individuals and coeval samples spanning the Mediterranean areas was employed to estimate the proportion of people buried in CB. As previously suggested [9,10], Eastern Mediterranean ancestry was also one of the leading components in the Roman Empire (Figure 3).

The cross-validation error gave a parameter useful for detecting the goodness-of-fit of the reconstruction: over five runs from K = 2 to K = 6, this parameter ranged from cv = 0.46491 for K = 3 to cv = 0.48458 for K = 6 (Supplementary Table S2). Accordingly, cv for the customized dataset suggests that K = 3 has the best fit for detecting people’s genetic compositions from CB. The leading genetic fraction was mostly shared with Eastern Mediterranean people (57.4%, in green in Figure 3, K = 3).

Two minor North Africa and European fractions (37.2%, in cyan, and 5.4%, in red in Figure 3, K = 3) could be scored. This signature green component was also shared by the coeval and topographically close Romans reported in Antonio et al. [9], whose green component related to the Eastern Mediterranean genomic fraction accounted for 61.1% (Figure 3). Remarkably, the frequencies of the other genomic components were switched between CB and other Romans from the study by Antonio et al. [9] and varied for the people buried in Quarto Cappello del Prete, a necropolis in the far eastern *Suburbium* of Rome [10].

The outgroup f3 statistic was calculated to understand the population’s genetic history and how two populations are genetically related. CB people were related to North African populations. We chose the Papuans as an outgroup to measure closer populations’ genetic relatedness from the outgroup f3 statistic compared with the two populations. Unsurprisingly, the CB genomic characteristics were closer to those of ancient Romans from Quarto Cappello del Prete [10]. However, the genomic similarity with other Romans is consistent with other ancient populations (Supplementary Figure S1). As previously noted, the genomic makeup of people inhabiting Rome and the neighbors differed from those of people from Roman Britain.

**Figure 4:**
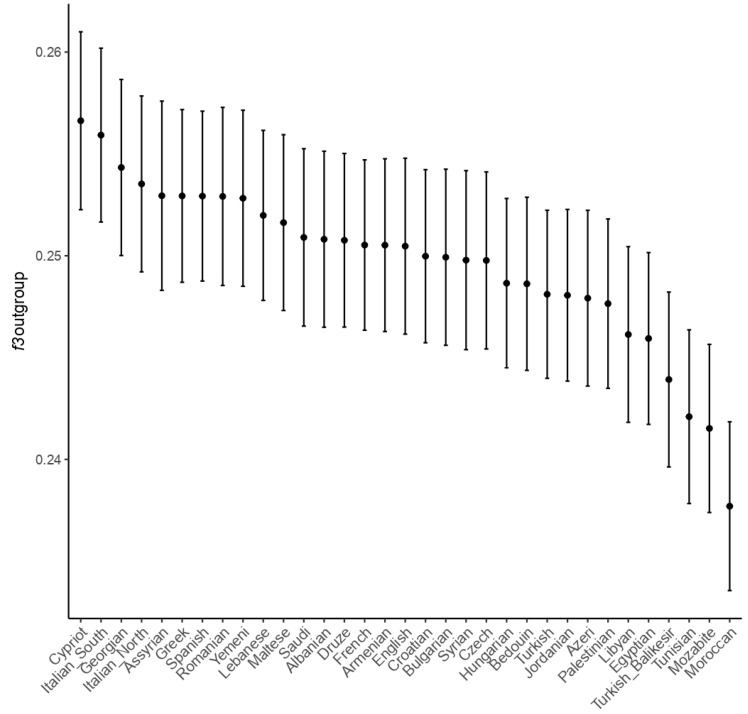
Visual representation of the f3-outgroup statistics for CB and present-day populations in the form of the f3 outgroup (Papuan; CB, populations).

The f3-outgroup comparison with present-day populations showed that the people buried in CB are more genetically similar to Europeans and Eurasians than people in the outgroup considered. This analysis pointed out the magnitude of the middle eastern genetic component previously mentioned (more positive f3-outgroup values). This finding mitigated the genetic similarity of CB with current North Africans, confined in the latest ranks of the plot, even though we should consider the tiny difference in the magnitude of the statistic (Figure 4) as well as the effect of the low sample size for the test sample. Indeed, as f3 statistics are defined as the product of allele frequency differences between populations, the small size of CB should affect the estimation, which should be considered with caution [53].

**Figure 5:**
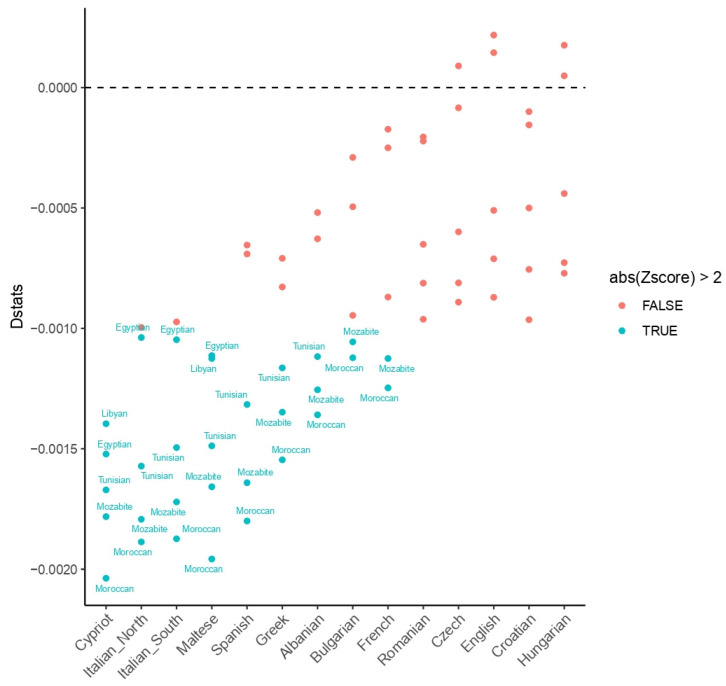
Visual summary of D-statistics of the form D (Papuan, CB; North Africans, Europeans). Dstats < 0: CB is more similar to the North African population in the label, Dstats > 0: CB is more similar to the European population in the *x*-axis. The significance of the analysis is expressed by abs (Zscore). Labels are reported only for significant analyses.

The D-statistic was also calculated as an additional parameter used to define correlations of allele frequency differences by evaluating a test population’s similarity for two references and an outgroup. Again, due to the influence of the North African genetic component across CB samples, the statistic was estimated considering Papuans as an outgroup, as we could deny their contribution to the gene flows among the populations tested. CB people were significantly more similar to North Africans than European and Middle Eastern individuals (Figure 5 and Supplementary Figures S2 and S3; Supplementary Table S2).

However, the already identified genetic legacy of ancient Romans with eastern populations is also significant. Even though we obtained reliable genetic information about only three individuals from CB, we can determine that they were all of the male sex (Supplementary Table S2). Accordingly, we leveraged the reads mapping on the Y chromosome to gather information about the Y-Chr haplogroups. Only two—T46 and F4_I—out of three individuals could be proficiently characterized to what concerns the male-specific uniparental marker. Both referred to Y-haplogroups consistent with the predominantly Southern European, Mediterranean, and Levant groups (J and a sub-clade of G2).

Conversely, the mitochondrial DNA haplogroups were identified for all three individuals (mtDNA coverage ranged from 44.64x to 91.33x), which again related to common European and Middle Eastern lineages such as X2n and H (Supplementary Table S2). The pairwise mismatch analysis able to identify whether genetic kinship relationships existed among these individuals was evaluated as the READ heuristic approach can successfully infer up to second-degree relationships with as little as 0.1x shotgun coverage. Accordingly, we performed this identity by state (IBS)-based analysis for T21, T46, and F4_I to identify previously unknown relationships among these peers even though their locations in the necropolis were different, confirming that the samples did not share familial legacy (Supplementary Figure S4).

### 3.2. Microbial Profiles

The evaluation of microbic genomes returned different metagenomic components for the three individuals. The bacterial clusters associated with T21 comprised species known to be putatively considered human pathogens (*Staphylococcus* sp., *Moraxella* sp., *Cutibacterium* sp.) along with microbes that are rarely reported to serve as pathogens but grow in soils contaminated with waste products (Table 3). No significant viral components were detected, while the protozoa characterized pertained to non-human parasites such as *Plasmodium coatenyi* and *Babesia bigemina*, even though most reads were related to *Toxoplasma gondii* (Supplementary Table S3 and Supplementary Figure S5).

Conversely, T46 showed many reads related to *Pseudomonas stutzeri*, a denitrifying bacterium widely distributed in the environment. Remarkably, some reads were mapped to *Fictibacillus arsenicus*, a Gram-positive arsenic-resistant bacterium able to grow in the presence of 20 mM arsenate. *Alcaligenes faecalis*, an apparently environmental saprophytic bacterium for which soft tissue infection is rare, was also identified [54]. Again, the *Clostridium botulinum* finding could be related to the soil: the species is not harmful, but they can produce toxins when deprived of oxygen, such as in stagnant soil or mud, which is likely present in burial environments [55] (Figure 3). No meaningful presence of *viridae* could be detected, while the protozoa analysis led us to consider the massive representation of *Toxoplasma gondii* (Supplementary Table S4 and Supplementary Figure S6).

Finally, the composition of microbial communities associated with F4_I was evaluated (Table 3). Again, no significant hits were found for viral and protozoic microbes; however, multiple soil-related bacteria were present. *Staphylococcus aureus* and *Glutamicibacter arilaitensis* were considered as they could also be recognized as opportunistic pathogens that could be responsible for human infections in the urinary tract [56,57]. Accordingly, they could be related to the *fullonica* environment (Supplementary Table S5 and Supplementary Figure S7) due to the massive amount of body waste.

The evaluations for T9 and F9 also returned species consistent with the sampling site. They confirmed the findings already reported for the specimens bearing a number of human reads suitable for downstream analysis. Indeed, the tooth specimen allowed us to detect the oral microbiome, supporting the quality of the estimation performed by HOME-BIO (Supplementary Table S6).

## 4. Discussion

Despite recent efforts to characterize the genomic makeup of ancient Romans [9,10], there is still a paucity of information about the genetic characteristics of a multifaceted environment such as that of the capital of the Roman Empire. Thus far, our knowledge about only a few individuals living—and dying—in the Urbs is limited. Furthermore, a multitude of different communities was sampled for genomic analysis. Sometimes, these human groups referred to specific social enclaves, whose genetic legacy could be misleading in the definition of the genetic characterization of ancient Rome. The genomic data of ancient Romans from Casal Bertone improve the genomic records for Imperial Age Romans (first–third centuries CE). Accordingly, our tiny survey will contribute to adding a piece of evidence about the multiethnic landscape characterizing one of the most densely populated urban centers from the past.

We recognize that the restricted sample size did not allow us to make population-based assumptions about all Roman inhabitants; however, our survey on a single necropolis was associated with a productive workshop—a *fullonica*, where people of low social strata and perhaps a humble background were permanently employed. They did not work pleasant jobs. In that workshop, clothes were sprinkled with ash and put into tubs filled with urine—a prime cleaning agent—or other alkaline substances used for cleaning. The *fullones* trampled the clothes to treat greasy stains with alkaline substances—such as the potassium hydroxide in ash or the ammonia in urine—to convert the grease into soluble salts of fatty acids.

Human or domestic urine was used for these characteristics, and often, the substance was publicly collected from the *latrinae* or public recipients. Its use was even taxed through the *vectigal urinae* tax enforced by Emperor Vespasian in the first century CE.

As depicted in one of the most renowned *fullonica* in Pompeii, after the clothes had been rinsed, they were placed over burning sulfur, and sulfur dioxide is a poisonous but effective bleaching agent. As can be easily argued, fulleries stank and were proverbially unpleasant places to be around [7]. The work was primarily performed by slaves, as reported by classical authors such as Varro.

Our genomic data support that certain ancestries characterized the people buried in CB. The occurrence of novel genetic contributions rather than the previously identified legacies [9,10] means that specific groups were responsible for the plant’s daily routine. Indeed, as for the previously analyzed samples from Rome, we can recognize a certain contribution of Eastern Mediterranean groups. However, CB individuals were genetically different from other individuals from Rome [9,10], even though we are aware that our approach could capture only partial information due to the restricted sample size. However, CB people were part of the same working community, while other samples referred to a collection of ancient communities. In that perspective, the heterogeneity of the larger comparative sample could hide the genetic characteristics of a single human group.

The evaluation of the putative gene flow between the CB community and present-day individuals underpins a shared allelic background with individuals living in the south shores of the Mediterranean Sea, as also partially confirmed by the admixture analysis.

Fulling was widespread in Greece as well as in the Roman Empire. However, classical Greek authors such as Aristophanes or Plato only casually referred to fullers as common figures without detailing their profession or socioeconomic role. Similarly, only a few iconographic representations were known, and only a tiny Hellenistic plant dating back to the first century BC was discovered at Delos [58]. However, some references point to *knapheis*—the Greek word behind fuller’s activity—in Egyptian papyri. This evidence is consistent with knowledge of fulling activity outside the Greek and Roman worlds, up until the southern coast of the Mediterranean basin.

In that perspective, the genetic background that we determined for people buried in Casal Bertone is remarkable. Indeed, they were young—and would not be allowed to be employed in the workshop—at least for T21 and T46, aged 6/9 months and 2 years ± 8 months, respectively. They were likely the offspring of people originating from North Africa and were already knowledgeable about fulling techniques. Conversely, F4-I was possibly already employed in the factory, as Roman children were put to work very early in life [59].

Indeed, most evidence for fulling is from many literary references dating back to the first three centuries CE. Papyri, wax tablets, lead tags, archaeological evidence, and iconographic depictions of fullers at work are widespread during the Imperial Age, coming from several locations in the Roman Mediterranean. However, three significant clusters of evidence were considered: Rome, Western Anatolia, and Egypt [7].

Remarkably, the three individuals from Casal Bertone pointed out genomic characteristics that fit these foci, suggesting a putative legacy with people living in areas where *fullonicae* and fullers were widely known, as also testified by records from Roman Egypt (P.Tebt. II, 40624).

Furthermore, the individuals analyzed in Casal Bertone could be considered offspring of people pertaining to one of the professional associations of skilled fullers known to be widespread in larger urban communities [6]. Indeed, guilds from Roman Italy, Roman Asia Minor, and Roman Egypt could act as bridges between fullers and local elites, representing the fullers’ interests to the local authorities [6,7]. Indeed, several epigraphic records were about how these associations buried their members or about certain social events that took place, i.e., a collegium of *fullones* involved in a struggle about their meeting place in Rome [7]. Again, papyri texts from Egypt reported about fullers’ associations in Oxyrhynchus and Tebtynis—160 km southwest of the city of Cairo and near the modern village of Tell Umm el-Baragat in the Al Fayyum Governorate in Lower Egypt, respectively—playing a significant role in tax collection for the municipalities to secure the economic interests of their members [6].

Thus far, the uniparental markers are consistent with a genetic origin with people spread throughout the Mediterranean area, even though they also stressed the near eastern origin reported for previously analyzed samples [9,10].

Remarkably, the diet preferences identified for people buried in Casal Bertone [14,19] suggested a peculiar diet for the Casal Bertone community, perhaps based on mixed consumption of C3 ecosystem-derived resources and aquatic resources. From that perspective, the difference we can recognize between CB and other coeval Roman communities could be consistent with cultural constraints due to different geographical origins and ethnicities.

The strontium and oxygen isotope analyses performed for CB people [22] already identified some people coming from a region with a hotter, drier climate than Rome. Even though other people could come from multiple areas to CB for employment, the consistency of the data suggests a North African origin, at least for some of them.

Furthermore, a mingled working environment constituted by people coming—as free or captive—from different areas subject to Roman Empire rule cannot be ruled out for Casal Bertone.

The evaluation of the microbes associated with the skeletal samples pointed out a constant presence of microbial communities reliably present in soils where massive contamination from body waste occurred, especially considering the use of urine. These findings are consistent with the environment in which the individuals were discovered.

Our data point to robust evidence that *Staphylococcus* sp. was widespread in the local environment. Indeed, multiple species of coagulase-negative staphylococci (*S. hominis*, *S. hemolyticus*, *S. epidermidis*, and *S. cohnii*) account for most urinary tract infections (UTIs) irrespective of gender [60,61,62]. Furthermore, a robust presence of bacterial genera suggestively related to the soggy setting of the plant was found: *Sphaerobacter* sp. and *Salinicoccus* sp. are commonly detected in sewage sludge [63] and wastewater also found in current textile plants [64]. T46 from area Q outlines a remarkable finding such as the *Fictibacillus arsenicus*, an arsenic-resistant microbe able to tolerate high concentrations of arsenic [65]. Notably, arsenic-based minerals such as orpiment and realgar were used to extract bride yellow sulfide from arsenic used for clothing dyes and to treat leather [66]. These dye compounds could be proficiently used in *fullonica* plants; thus, the presence of an arsenic-tolerant species such as *F. arsenicus* is highly consistent with the environmental microbiota. Finally, other environmental genera could be meaningful for depicting workplace environmental biotic factors. Multiple environmental species related to wastewater and sewage sludge could be detected, such as *Sphaerobacter* sp. and *Salinicoccus* sp.

Overall, the samples show that the tannery wastewaters could have caused severe sanitary damage for people who lived and worked nearby, with striking comparisons with evidence from currently dyeing vats in Chouara, Morocco [67]. This tannery still runs and represents one of the most iconic locations in Fez city, where the ancient craft of tanning and dyeing, even still using cow urine and pigeon manure for the process, is still performed. A biomolecular analysis of its wastewater accounting for the microbic communities revealed the presence of toxic elements strongly affecting the water quality. However, the results were barely comparable with the toxins of ancient tanneries [67]. A similar scenario was also recognized for Indian tanneries [68].

We are aware that the bone districts submitted to the analysis were not indefectible areas where a significant metagenomic survey could be outlined. However, due to the still ongoing osteological evaluation, we were granted access to these exploratory samples, from which the results could be considered inductive for future ad hoc designed comprehensive assessments.

## 5. Conclusions

This survey outlines the genomic legacy of people employed in the most extensive Roman tannery. Despite *fullones* being renowned for many inscriptions from Italy and the Roman Empire, the biological and genomic characterization of fullers are mostly unknown. We generated reliable whole-genome data for three individuals buried in Casal Bertone, pointing out their North African genomic makeup. This evidence is consistent with the already known prominence of tanning activities in the southern shores of the Mediterranean basin, where a tannery similar to the one found in Casal Bertone still runs. To the best of our knowledge, the present analysis is the first to consider individuals related to these workshops. Our findings support the hypothesis that knowledgeable individuals who were putative heirs of people coming from areas where the activity was steadily implemented were employed in these areas. Remarkably, this resolution was also consistent with the isotopic evidence [3,14,19]. Due to the peculiar environment, we completed the first analysis of microbial communities detectable in a shotgun analysis. We confirmed the risky environment of the Casal Bertone area, which was already suggested by osteological comments [14,15,20,21], which already paved the way for considering the presence of infectious diseases. Despite the tiny sample size, our analysis contributes to broadening the knowledge about the genomic characterization of Imperial Rome, which is increasingly characterized by multiple ancestries that were only described by text and iconographic sources up until recently. The broadening of the sample to be submitted to genomic and isotopic analyses is meaningful in supporting our considerations and making an even more thorough reconstruction of the bio-cultural characteristics of the inhabitants of one of the most influential cities from the past.

## Figures and Tables

**Table 1:** Samples’ characteristics.

Sample	Age	Area	Skeletal District
T9	18 months ± 3	Necropolis	Petrous bone
T21	6/9 months	Area Q	Petrous bone and femur
T46	2 years ± 8 months	Area Q	Petrous bone
F4_I	4/9 years	Mausoleum	Petrous bone and femur
F9	9/15 years	Mausoleum	Femur and tooth

**Table 2:** Summary table for the individuals suitable for the analyses.

Sample Name	Number of Reads	Coverage (Mean)	Coverage (SD)	Non-Missing Calls vs. 1240 K Panel
T21	2,342,021	0.0562	0.2982	31,972
F4_I	2,029,640	0.0458	0.2662	27,178
T46	1,267,711	0.0311	0.2228	17,522

**Table 3:** Summary table for the top 15 hits for the bacterial organisms identified in the analysis.

Reads to This Taxon	NCBI ID	Indented Scientific Name
**T21**		
12,050	511	*Alcaligenes faecalis*
6096	507	*Alcaligenes*
2482	255247	*Fictibacillus arsenicus*
2031	110319	*Nocardioides* sp. *CF8*
1803	929704	*Myroides odoratus DSM 2801*
1182	1485	*Clostridium*
969	1329200	*Fictibacillus*
886	286	*Pseudomonas*
878	1386	*Bacillus*
715	1471761	*Novibacillus thermophilus*
696	292459	*Symbiobacterium thermophilum IAM 14863*
642	1123519	*Pseudomonas stutzeri DSM 10701*
637	269800	*Thermobifida fusca YX*
560	717605	*Thermobacillus composti KWC4*
559	1491	*Clostridium botulinum*
**T46**		
13,362	1873	*Micromonospora*
10,458	286	*Pseudomonas*
9684	1123519	*Pseudomonas stutzeri DSM 10701*
5943	129337	*Geobacillus*
4711	255247	*Fictibacillus arsenicus*
3250	1883	*Streptomyces*
1862	613	*Serratia*
1804	1329200	*Fictibacillus*
1349	1007105	*Pusillimonas* sp. *T7-7*
1259	1471761	*Novibacillus thermophilus*
1244	1491	*Clostridium botulinum*
1078	316	*Pseudomonas stutzeri*
990	1747	*Cutibacterium acnes*
976	511	*Alcaligenes faecalis*
883	1196031	*Bacillus oceanisediminis 2691*
**F4_I**		
750	286	*Pseudomonas*
455	1279	*Staphylococcus*
385	1280	*Staphylococcus aureus*
310	469	*Acinetobacter*
291	382	*Sinorhizobium meliloti*
251	479434	*Sphaerobacter thermophilus DSM 20745*
181	305	*Ralstonia solanacearum*
174	407035	*Salinicoccus halodurans*
173	1485	*Clostridium*
170	358	*Agrobacterium tumefaciens*
165	861360	*Glutamicibacter arilaitensis Re117*
144	1386	*Bacillus*
114	357	*Agrobacterium*
114	1712675	*Turicibacter* sp. *H121*
111	1301	*Streptococcus*

## Data Availability

The data that support the findings of this study are available from the corresponding authors upon reasonable request.

## Supplementary Materials

The following supporting information can be downloaded at: https://www.mdpi.com/article/10.3390/genes13010136/s1, Supplementary Table S1: Reference populations used for the comparisons; Supplementary Table S2: D-statistics and Z-score as D (Papuan, CB; Pop1, Pop2); Supplementary Table S3: Microbial findings for T21; Supplementary Table S4: Microbial findings for T46; Supplementary Table S5: Microbial findings for F4_I; Supplementary Table S6: Microbial findings for T9 and F9; Supplementary Figure S1: Visual representation of f3-outgroup statistics for CB and ancient populations in the form of f3-outgroups (Papuan; CB, Populations); Supplementary Figure S2: Visual summary of D-statistics of the form D (Papuan, CB; Asians, Europeans). Dstats < 0: CB is more similar to the Asian population in the label, Dstats > 0: CB is more similar to the European population in the *x*-axis. The significance of the analysis is expressed by abs (Zscore). Labels are reported only for the significant analysis; Supplementary Figure S3: Visual summary of D-statistics of the form D (Papuan, CB; North Africans, Asians). Dstats < 0: CB is more similar to the North African population in the label, Dstats > 0: CB is more similar to the Asian population in the *x*-axis. The significance of the analysis is expressed by abs (Zscore). Labels are reported only for significant analyses; Supplementary Figure S4: Visual kinship reconstruction for the pairs reported in the *x*-axis; Supplementary Figure S5: Visual representation of the bacterial findings associated with T21; Supplementary Figure S6: Visual representation of the bacterial findings associated with T46; Supplementary Figure S7: Visual representation of the bacterial findings associated with F4_I.
