# Supplementary material for: First Glimpse into the Genomic Characterization of People from the Imperial Roman Community of Casal Bertone (Rome, First–Third Centuries AD)
Source: Genes (Basel). 2022 Jan 13;13(1):136. doi: 10.3390/genes13010136 (PMC8774527; doi:10.3390/genes13010136)
Supplement: Supplementary file 1 [file genes-13-00136-s001.zip › Supplementary Figures S1-S7.pdf]

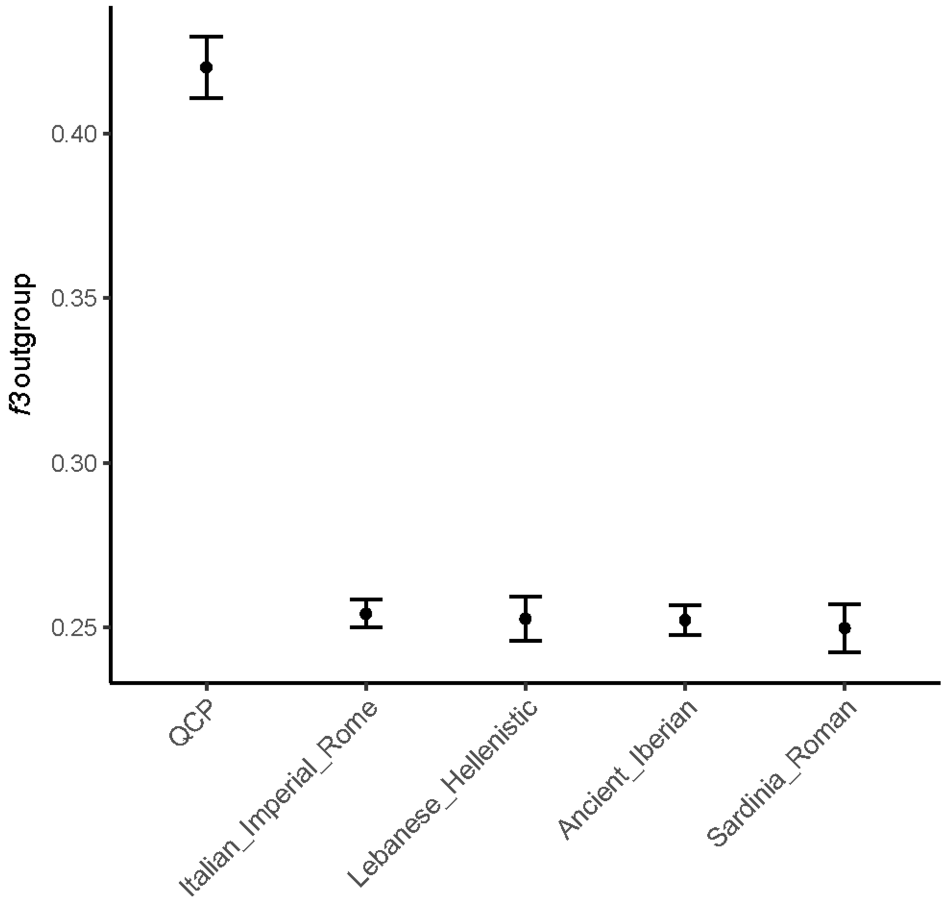

Supplementary Figure S1: Visual representation of f3-outgroup statistics for CB and ancient populations in the form of f3-outgroups (Papuan; CB, Populations).

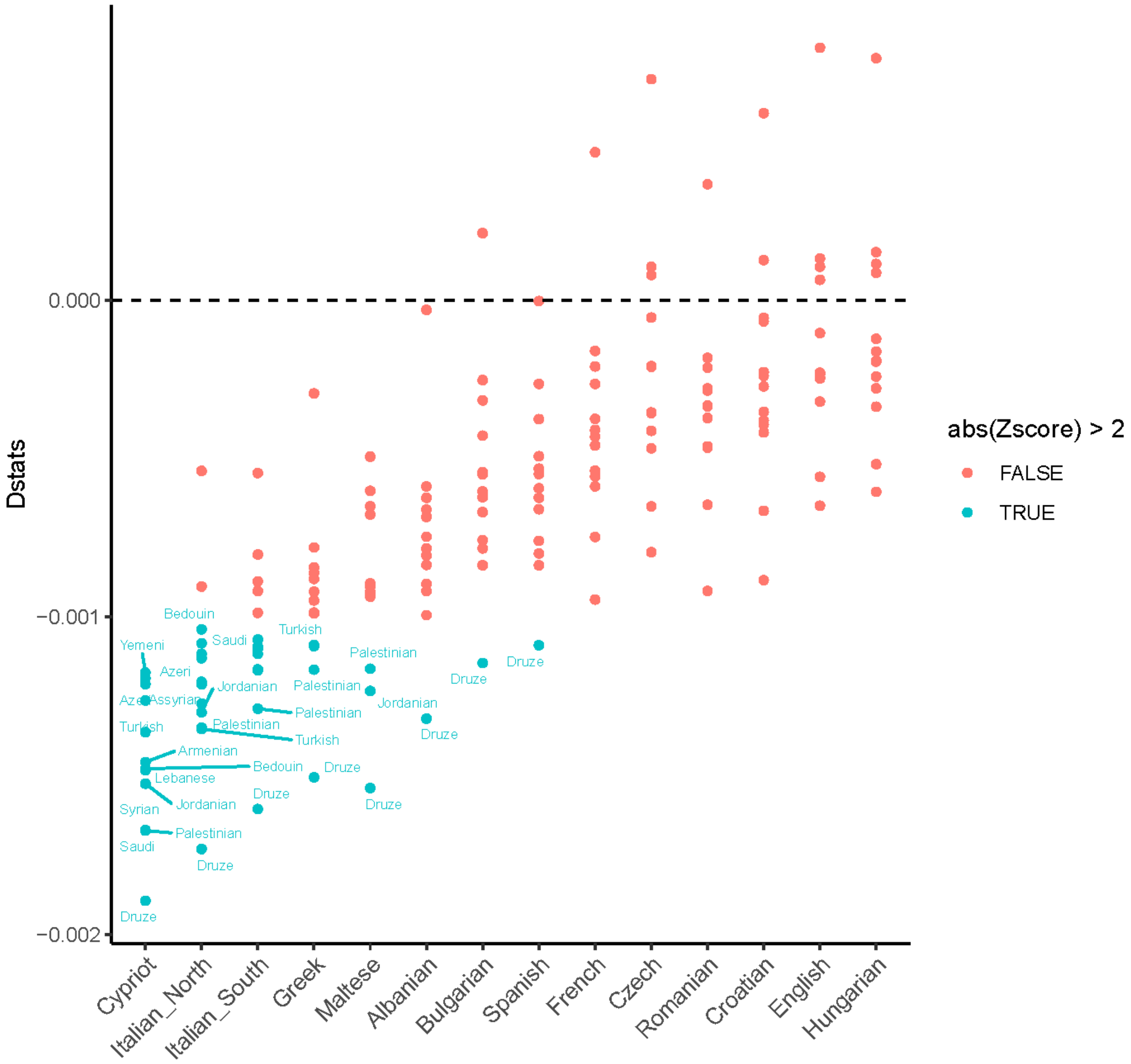

Supplementary Figure S2: Visual summary of D-statistics of the form D (Papuan, CB; Asians, Europeans). Dstats < 0: CB is more similar to the Asian population in the label, Dstats > 0: CB is more similar to the European population in the *X*-axis. The significance of the analysis is expressed by abs(Zscore). Labels are reported only for the significant analysis.

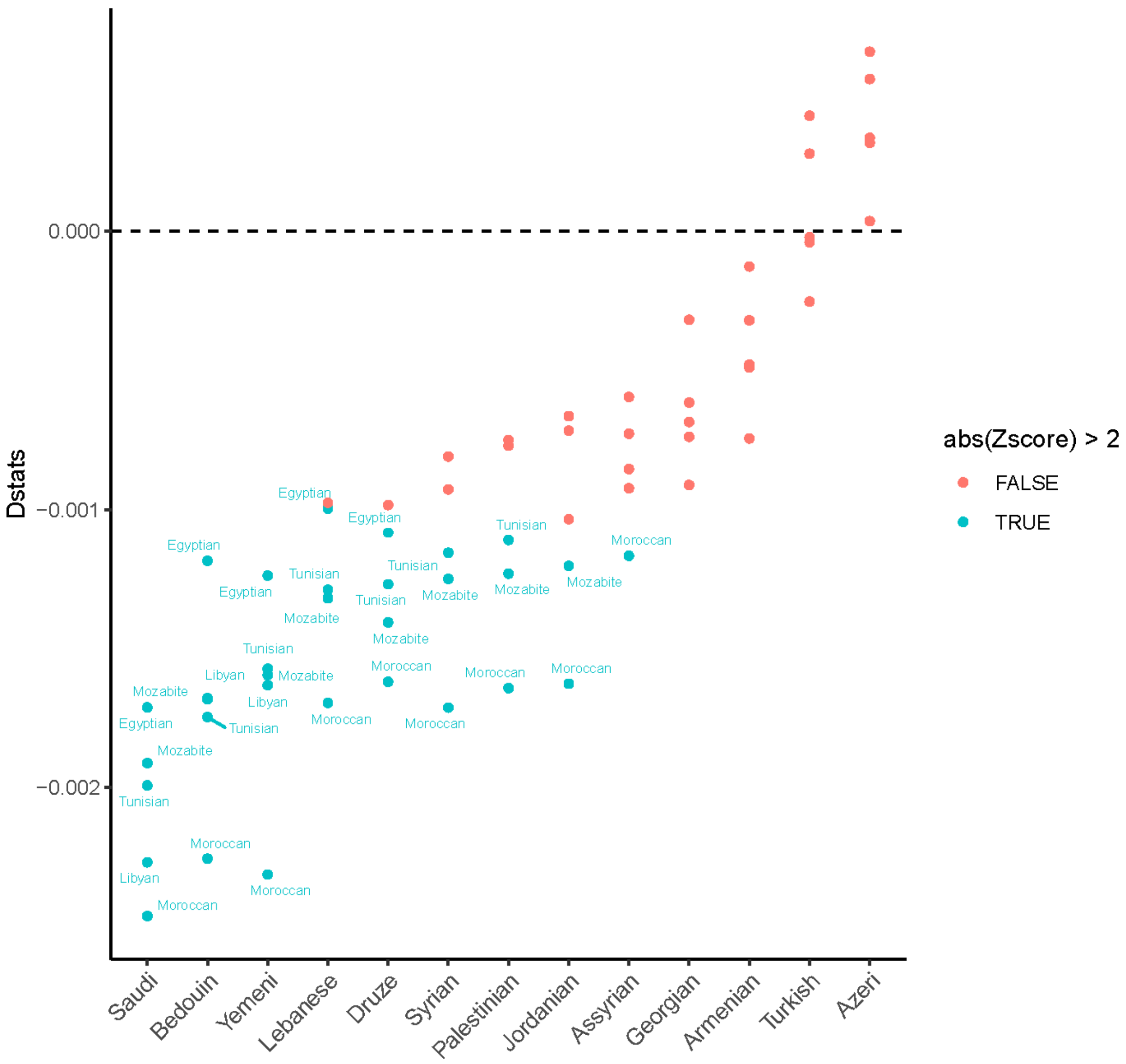

Supplementary Figure S3: Visual summary of D-statistics of the form D (Papuan, CB; North Africans, Asians). Dstats < 0: CB is more similar to the North African population in the label, Dstats > 0: CB is more similar to the Asian population in the *X*-axis. The significance of the analysis is expressed by abs (Zscore). Labels are reported only for significant analyses.

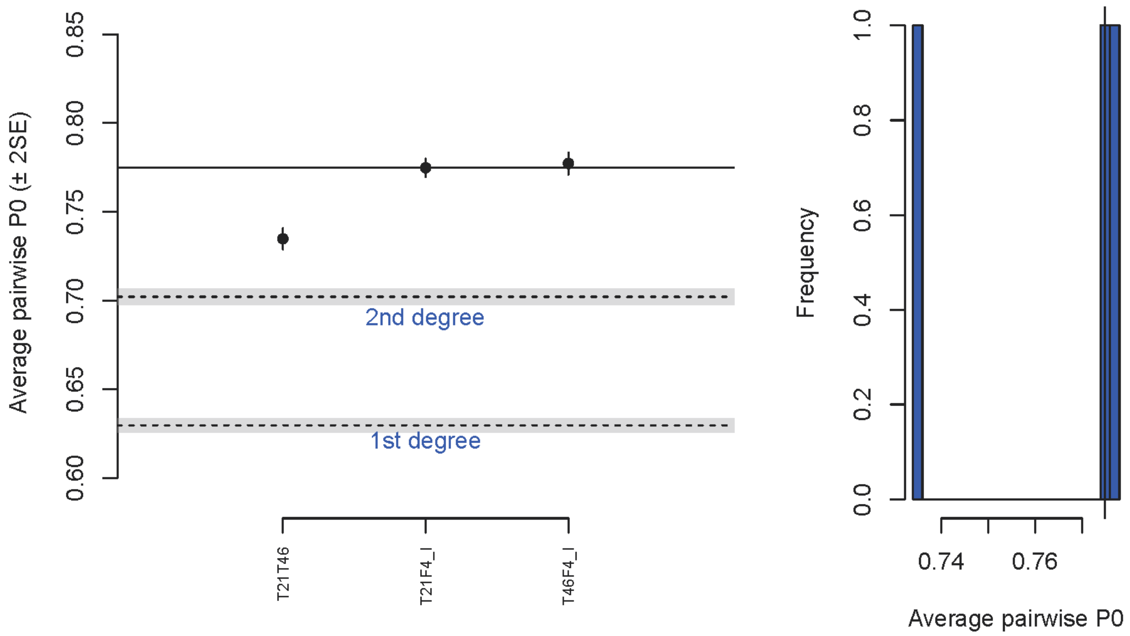

Supplementary Figure S4: Visual kinship reconstruction for the pairs reported in the *X*-axis.

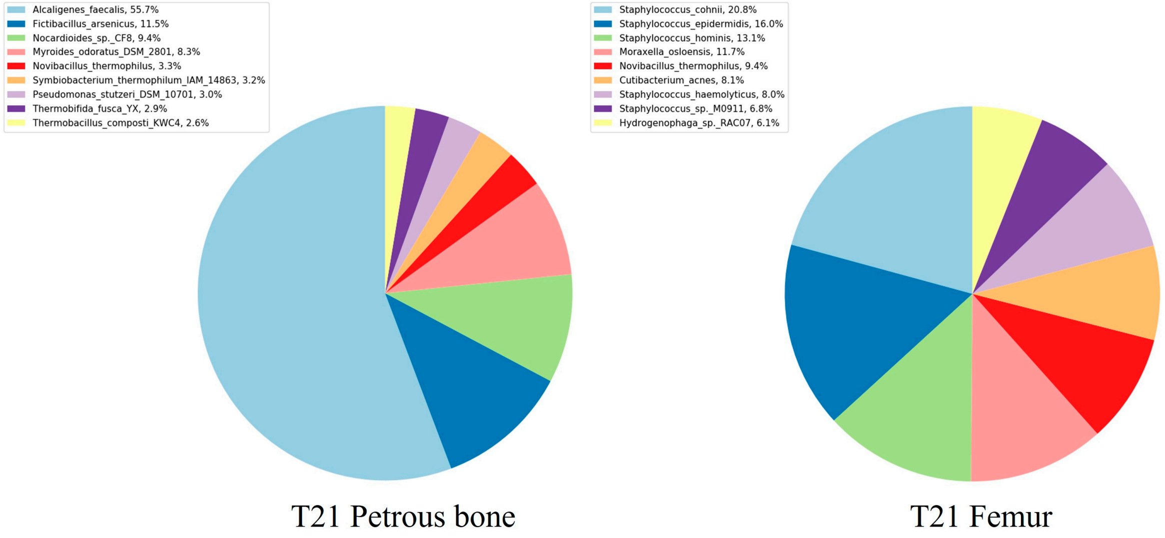

Supplementary Figure S5: Visual representation of the bacterial findings associated with T21.

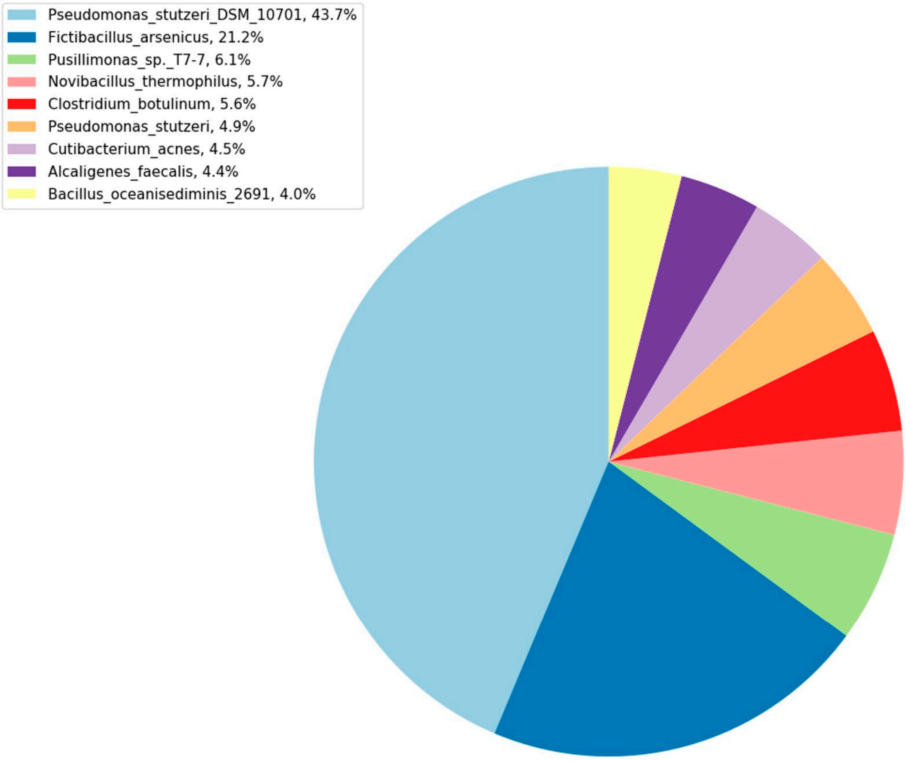

T46 Petrous bone

Supplementary Figure S6: Visual representation of the bacterial findings associated with T46**.**

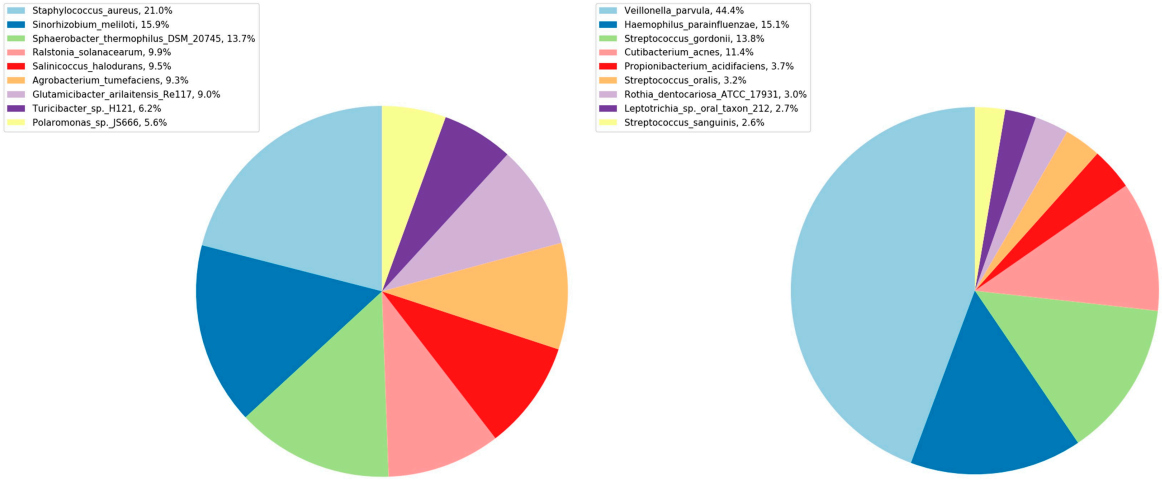

F4_I Petrous bone

F4_I Femur

Supplementary Figure S7: Visual representation of the bacterial findings associated with F4_I.
